# Life in a harsh environment: the effects of age, sex, reproductive condition, and season on hair cortisol concentration in a wild non-human primate

**DOI:** 10.7717/peerj.9365

**Published:** 2020-06-23

**Authors:** Paul A. Garber, Anna McKenney, Evelyn Bartling-John, Júlio César Bicca-Marques, María Fernanda De la Fuente, Filipa Abreu, Nicola Schiel, Antonio Souto, Kimberley A. Phillips

**Affiliations:** 1 Department of Anthropology and Program in Ecology, Evolution, and Conservation Biology, University of Illinois, Urbana, IL, USA; 2 Natural Resources and Environmental Sciences, University of Illinois, Urbana, IL, USA; 3 Department of Psychology, Trinity University, San Antonio, TX, USA; 4 Escola de Ciências da Saúde e da Vida, Laboratório de Primatologia, Pontifícia Universidade Católica do Rio Grande do Sul, Porto Alegre, RS, Brazil; 5 Departamento de Biologia, Laboratório de Etologia Teórica e Aplicada, Universidade Federal Rural de Pernambuco, Recife, PE, Brazil; 6 Departamento de Zoologia, Laboratório de Etologia, Universidade Federal de Pernambuco, Recife, PE, Brazil; 7 Southwest National Primate Research Center, Texas Biomedical Research Institute, San Antonio, TX, USA

**Keywords:** Common marmosets, *Callithrix jacchus*, Environmental stress, Cortisol, Caatinga

## Abstract

Hair cortisol concentration (HCC) provides a long-term retrospective measure of hypothalamic–pituitary–adrenal axis activity, and is increasingly used to assess the life history, health and ecology of wild mammals. Given that sex, age, season and pregnancy influence HCC, and that it may indicate ongoing stress, we examined HCC in common marmosets (*Callithrix jacchus*) naturally inhabiting a hot and dry semi-desert like habitat, Caatinga, in northeastern Brazil. We trapped, measured, weighed, marked and collected shaved hair from the back of the neck of 61 wild marmosets during the wet and dry seasons. Using enzyme immunoassay, we found that HCC was higher in the dry season compared with the wet season among all age/sex classes. Females had significantly higher HCC than males, juveniles had higher HCC than adults, and reproductively active adult females and non-pregnant/non lactating adult females did not differ in HCC. There were no interaction effects of sex, age, group, or season on HCC. The magnitude of the effect of this extremely hot and dry environment (average yearly rainfall was only 271 mm) on HCC in common marmosets is difficult to ascertain as these animals are also experiencing a variety of other stressors. However, the elevated HCC seen in common marmosets during the 5–8 month dry season, suggests these primates face an extended period of heat, water and possibly nutritional stress, which appears to result in a high rate of juvenile mortality.

## Introduction

Glucocorticoid hormones, of which cortisol is the most prominent in primates, are involved in multiple physiological processes, including the conversion of sugar, fat and protein stores into usable energy, and as an anti-inflammatory preventing tissue and nerve damage (Katsu & Iguchi, 2016). Cortisol is regulated by the hypothalamic–pituitary–adrenal (HPA) axis and its production is increased during periods of stress and elevated blood glucose levels. Environmental stressors such as habitat fragmentation (Seltmann et al., 2017), climate extremes (Bechshoft et al., 2013; Fardi et al., 2018), reduced food availability and anthropogenic disturbances (Carlitz et al., 2016; Strasser & Heath, 2013) impact HPA-axis activity. The implications of such prolonged stress may include decreased fertility (Strasser & Heath, 2013), a weakened immune system (Khansari, Murgo & Faith, 1990; Padgett & Glaser, 2003) and decreased body condition (McEwen, 1998), which can lead to increased mortality.

Hair cortisol concentration (HCC) is increasingly used as a noninvasive measure to assess retrospective HPA axis activity in wild animal populations (Heimburge, Kanitz & Otten, 2019). While the exact mechanism by which cortisol is deposited into hair is unknown, free cortisol is believed to be incorporated into hair via diffusion from follicular capillaries into the medulla of the hair shaft during growth (Meyer & Novak, 2012; Russell et al., 2012). HCC reflects HPA axis activity over a period of weeks to months, depending on the rate of hair growth and the length of hair sampled. However, due to wash out effects, HCC is best used to evaluate ongoing stress, rather than a specific event that occurred in the distant past (Kalliokoski, Jellestad & Murison, 2019). That is, if individuals encounter environmental, nutritional, or social stressors that persist for an extended period, HCC can provide useful information about physiological response and physiological condition.

Environmental stressors including reduced food availability, drought, and exposure to excessive heat and water deprivation have been correlated with elevated cortisol levels in several animal species (Bruener, Delehanty & Boonstra, 2013; Cavigelli, 1999; Fardi et al., 2018; Parker et al., 2003; Weingrill et al., 2004). For example, a decrease in ripe fruit production was the best predictor of increased fecal cortisol levels in wild red-bellied lemurs (*Eulemur rubriventer*; Tecot, 2013). In the case of brown bears (*Ursus arctos*; Cattet et al., 2014) inhabiting Coastal British Columbia, an increase in salmon availability and consumption resulted in a decrease in HCC (Bryan et al., 2013). And, in Merino sheep (*Ovis aries*), elevated plasma cortisol levels were associated with a “loss of body water in excess of that associated with a loss of electrolytes”, diuresis and dehydration (Parker et al., 2003). This also was supported in a study of corridale sheep (*Ovis aries*) exposed to alternative regimes of heat stress and water restriction. Ewes exposed to a temperature–humidity index of 27.9 (mild stress), and water restriction for the first 3 h after feeding, exhibited significantly higher wool cortisol levels than ewes with unrestricted or less restricted (2 h post feeding) access to water (Ghassemi Nejad et al., 2014). Long-term exposure to heat, water and nutritional stress can result in the impairment of ovarian function, a decrease in rates of conception, reduced growth, dehydration, increased susceptibility to disease and death (Sanin, Zuluaga Cabrera & Tarazona Morales, 2015; Takahashi, 2012).

HCC is reported to display an age-dependent decline from young to adult ages in captive and domesticated species, including dairy cattle (*Bos taurus*), rhesus monkeys (*Macaca mulatta*), and vervet monkeys (*Chlorocebus aethiops*) (Dettmer et al., 2014; Fourie & Bernstein, 2011; Gonzalez-De-la-Vara et al., 2011; Laudenschlager, Jorgensen & Fairbanks, 2012). This same effect has been documented in wild primates such as vervets and baboons (*Papio* spp; Fourie et al., 2015a, 2015b). Similarly, blood glucocorticoids were reported to be higher in captive infant and juvenile pig-tailed macaques (*Macaca nemestrina*) compared with adults. This may result from infants and juveniles having lower corticosteroid binding globulin concentration, leading to higher plasma concentrations of free cortisol, which could be deposited into hair during growth (Grant et al., 2017).

There are inconsistent results concerning whether HCC varies by sex in nonhuman primates. Phillips et al. (2018) did not detect significant sex differences among captive adult common marmosets (*C. jacchus*). Similarly, researchers did not find sex differences in HCC in captive chimpanzees (*Pan troglodytes*) and orangutans (*Pongo* spp.) (Carlitz et al., 2014; Yamanashi et al., 2013). However, females were reported to have higher HCC compared to males in captive vervet monkeys (Laudenschlager, Jorgensen & Fairbanks, 2012). This sex difference emerged at puberty and continued into adulthood (Laudenschlager, Jorgensen & Fairbanks, 2012). Finally, wild adult female and male lion tamarins (*Leontopithecus rosalia*) showed similar values of fecal cortisol, except during the final trimester of pregnancy, when values for breeding females increased significantly (Bales et al., 2005, 2006).

In this study we assess the effects of age, sex, reproductive condition and season on HCC in a wild New World primate, the common marmoset (*C. jacchus*, Callitrichinae). Common marmosets are small monkeys (adult body mass = 265−325 g; Garber et al., 2019) endemic to forested habitats of two highly distinct biomes in northeastern Brazil:the Caatinga (CAT) and the Atlantic Forest (AF) (Garber et al., 2019; Rylands, Coimbra-Filho & Mittermeier, 2009). The CAT is a semi-desert biome characterized by a hot (daytime temperatures in the dry season commonly exceed 33 °C) and extended (5–8-month-long) dry season, limited rainfall (250–1,200 mm per year), drought-resistant plant species, and reduced productivity compared with the AF, which is a region of high biodiversity, higher annual rainfall (1,400–2,000 mm per year) and high primary productivity (Araújo, Castro & Alburquerque, 2007). Based on biogeographical and genetic evidence, it appears that ancestral common marmosets first invaded a Caatinga-like biome some 800,000 years ago (Buckner et al., 2015).

Marmosets (and their close relatives the tamarins) exhibit a highly derived set of reproductive traits that distinguish them from other primate taxa (Garber et al., 2016; Schiel & Souto, 2017). These traits include the production of dizygotic twin offspring, the ability to produce two sets of twin litters per year, and a system of cooperative infant caregiving provided principally by adult males (Garber et al., 2016). Adult males carry, provision and guard the group’s infants (Garber, 1997). Studies of several tamarin and marmoset species indicate a significant positive relationship between the number of adult male helpers per group and infant survivorship (Garber, 1997; Koenig, 1995).

Common marmosets live in small multimale–multifemale social groups composed of 5–16 individuals (Garber et al., 2019; Yamamoto et al., 2009). Wild groups contain from one to six adult females and one to five adult males. Regardless of the number of adult females per group, generally only a single female in each group breeds (Garber et al., 2019; Schiel & Souto, 2017). Reproductive competition among resident females for the sovereign or primary breeding position is high (Garber, 1997), and the breeding female is socially dominant to all other group members (De la Fuente et al., 2019). In both the AF and the CAT, a breeding female can produce two litters or a total of four offspring per year (Garber et al., 2019).

Given that the CAT represents an extreme environment characterized by high heat and water stress and reduced plant productivity, especially during the extended dry season, we examined and compared HCC, an indicator of overall health and HPA axis activity, among breeding female, non-breeding adult female, adult male and juvenile CAT common marmosets. Based on the existing literature, we hypothesized that juveniles would have higher HCC than adults (age effect) and that marmosets, regardless of age or sex, would show higher HCC during the dry season compared to the wet season. Given the joint role that mothers and helpers play in infant care giving, we did not expect differences in HCC between adult males and females; however, given the high nutritional costs of producing twin infants, we expected breeding females to experience increased HCC compared to non-breeding females. Finally, assuming that juvenile marmosets are more susceptible to environmental stressors (climate, access to resources, predation) than adults, we expected high rates of juvenile mortality in our study population.

## Method

From February 2015 to July 2018, we trapped, measured, weighed, marked and collected shaved hair from the back of the neck of 61 common marmosets belonging to ten groups inhabiting the Baracuhy Biological Field Station (7°31′42″S, 36°17′50″W) in the state of Paraíba in northeastern Brazil. The field site is a 400-ha thorn scrub Caatinga forest averaging 337 mm of rainfall per year (based on 85 years of data collected by the Instituto Nacional de Meteorologia-INMET). During the 3 years of our study, rainfall averaged 271 mm (SD ± 124 mm) and temperatures reached or exceeded 33 °C (91 °F) on 169 days (± 38) per year (based on an average of 330 days of data collection per year, INMET), making this one of the driest and hottest field sites inhabited by any species of nonhuman primate.

The monkeys were trapped using the Peruvian Capture Method, which involved habituating each marmoset group to a single large trap containing 10 separate compartments, each with its own individually operated door (Garber et al., 2019, for additional information). The traps were baited with bananas and during each capture session we were able to trap all or most group members. Once the group was trapped, we removed one marmoset at a time, injected the individual with ketamine HCL (50 mg/mL, dosage for juveniles = 0.02 mg and dosage for subadults and adults = 0.04 mg), collected biomedical information, and obtained a shaved hair sample from the back of the animal’s neck. The hair was immediately placed in a paper envelope, labeled with the marmoset’s identification number, age, sex and date of capture, and stored in a dry container at the field site. Marmoset hair samples were collected in the dry season (February/March) and in the wet season (July/August). Given that HCC is an indicator of stress occurring over a period of weeks or months, our trapping procedure is not expected to have a direct effect on our results.

In order to avoid resampling hair from the same individual during the same trapping season, all trapped individuals were implanted subcutaneously with an RFID microchip for permanent identification (Biomark HPT8 pit tags). During retrapping, we confirmed the identify of each individual using a microchip reader (Biomark 601 Handheld Reader). In addition, adults were fitted with a uniquely color-coded collar for field identification. Juveniles were too young to collar and instead a segment of their tail was shaved for purposes of field identification. Thus, we are confident that we did not collect duplicate samples from the same individual during the same trapping season of the same year. We sampled a total of 61 marmosets: 40 in the wet season and 26 in the dry season. Duplicate samples were obtained from three individuals. One marmoset was sampled twice (wet and dry season), and two individuals were sampled three times (twice in two distinct dry seasons, once in the wet season). We treated these samples as independent for analysis because they were collected between 4 and 24 months apart. Based on the length of the shaved hair samples (12–14 mm) used for analysis, and the fact that hair growth in captive marmosets is approximately .5 cm per month (Phillips et al., 2018), we estimate that the HCC represents marmoset physiology during the preceding 8–12 weeks.

Individuals were categorized as infant (≤4 months of age), juvenile (>4–11 months of age, subadult (12–15 months of age) or adult (>15 months of age) based on body mass, limb, body and genital measurements, and patterns of deciduous and permanent dental eruption (Hershkovitz, 1977). Adults were defined as reproductively mature individuals with all permanent dentition fully erupted (mean weight of adult males and females was 280 g; Garber et al., 2019). Subadults were individuals who had not attained full adult body mass (mean weight of subadults (*n* = 4) was 234 g) and without canines fully erupted. Juveniles were defined as individuals with a mixture of deciduous and permanent teeth, first permanent molar fully erupted and permanent canine had not yet erupted (Hershkovitz, 1977). Mean juvenile male and female body mass was 162.5 g, *n* = 21). Infants were not used in this analysis. Although marmoset groups in our population contained from one to four adult females, only one female in each group produced offspring. Adult females were classified as either pregnant/lactating or nonpregnant/nonlactating at the time of capture.

In addition, we conducted field censuses every 2–3 months via behavioral observations and occasional retrapping of 6 focal study groups in order to assess changes in their size, age and sex composition due to births, immigration/emigration, and disappearances. We used this information to obtain estimates of infant mortality (these results were published in Garber et al., 2019) and presumed juvenile mortality (i.e., the absence of juveniles previously present in the group). Given that it was not possible to obtain complete counts of all groups during each census, our data do not permit us to determine the degree to which season had an affect on juvenile mortality.

Permission to trap and collect biological samples from the marmosets was provided by the Federal Rural University of Pernambuco, Brazil (CEUA license number 135/2014 and SISBIO license number 46770-1). Permission to collect behavioral data on the marmosets was approved by the The University of Illinois (IACUC Protocol #14263).

### Hair cortisol concentration

Hair samples were stored at ambient temperature out of direct light in opaque paper envelopes until processed. Stored under these conditions, hair cortisol is stable (Greff et al., 2019; Yamanashi et al., 2016).

The protocol for extracting cortisol from hair followed Phillips et al. (2018), which was based on a procedure developed by Meyer et al. (2014). After processing, samples were diluted with PBS 1:40 before being analyzed in duplicate via enzyme immunoassay (EIA) using a commercially available expanded range high sensitivity salivary cortisol kit (#1-3002; Salimetrics, State College, PA, USA). Details of the dilution determination and parallelism tests for validation of the procedure for common marmosets are described in Phillips et al. (2018). We converted the obtained values (μg/dL) to pg/mg for analysis. Inter- and intra-coefficients of variance were 11% and 5%, respectively.

### Data analysis

We used ANCOVA to determine if there was a significant effect of (a) sex, age class, or season on HCC controlling for body mass and (b) reproductive condition (reproductively active or not reproductively active) and season on HCC. Adult females who had given birth within 1 month of hair sample collection and lactating or were pregnant as indicated by uterine palpation during the physical exam at the time of sample collection were classified as reproductively active. Analyses were conducted in SPSS 26.0 and α was set at 0.05.

## Results

**Figure 1:**
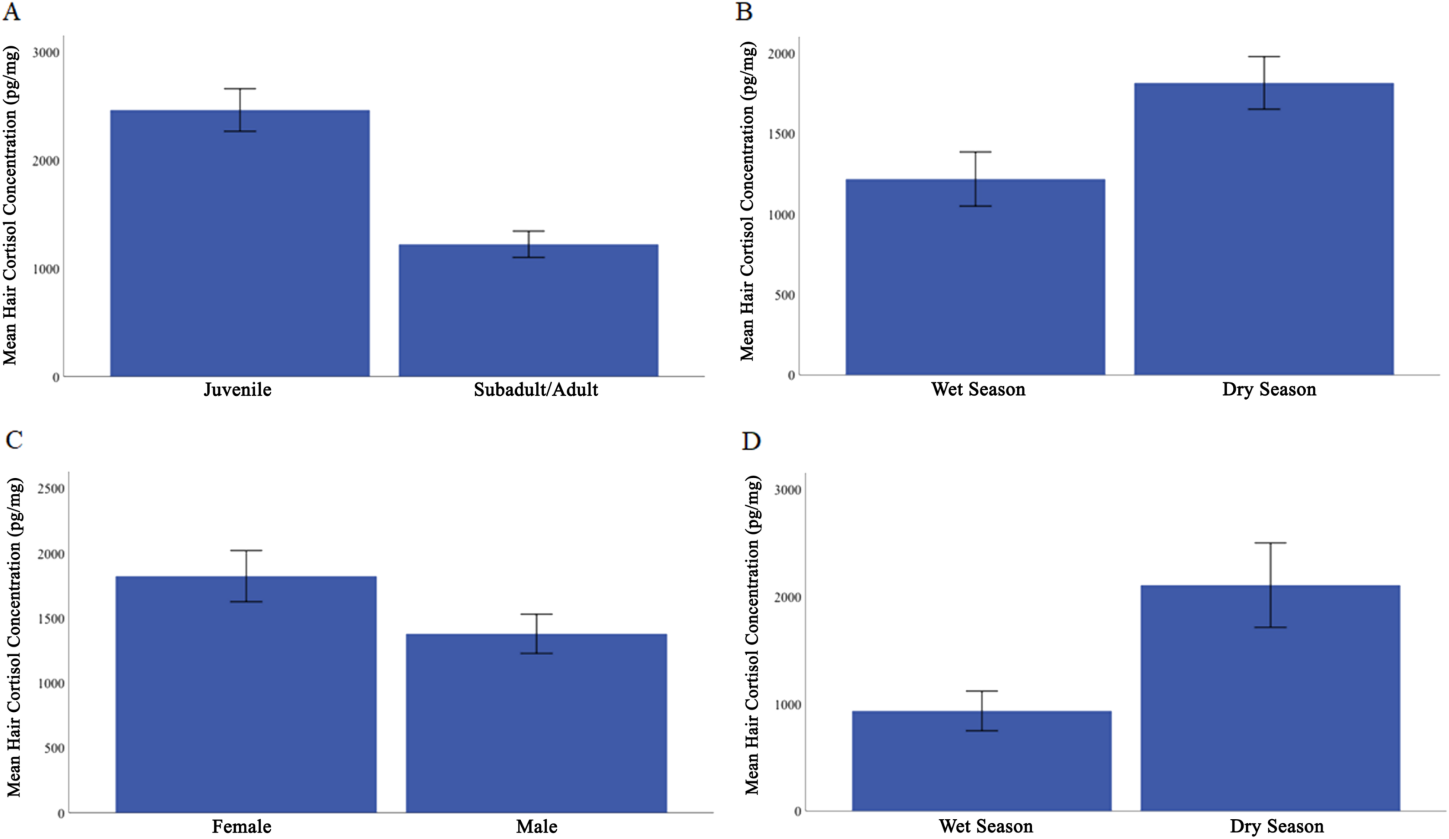
Mean hair cortisol concentration from Caatinga common marmosets. (A) Juvenile and subadult/adult. (B) All individuals during the wet season compared with dry season. (C) Females compared with males. (D) Adult females during the wet and dry seasons. Error bars represent ± 1 standard error.

Overall, HCC ranged from 231 to 4295 pg/mg (Table 1). We found that the covariate, body mass, was not significantly related to HCC, *F* (1, 57) = 0.070, *p* = 0.792, η_p_^2^ = 0.001. The predicted main effects of age class and season were significant (age class: *F* (1, 57) = 8.604, *p* = 0.005, η_p_^2^ = 0.131; season: *F* (1, 57) = 5.449, *p* = 0.023, η_p_^2^ = 0.087). Juveniles (*M* = 2464.71, SE = 196.54) had higher HCC than did subadults and adults (*M* = 1222.00, SE = 121.23; Fig. 1A). Across all individuals, HCC was higher in the dry season (*M* = 1816, SE = 164) than in the wet season (*M* = 1218, SE = 168; Fig. 1B).

**Table 1:** Hair cortisol concentration (HCC) in adult and juvenile female and male common marmosets during the wet and dry seasons in the Caatinga forest of northeastern Brazil. Adult females were assigned to pregnant/lactating (P/L) or nonpregnant/nonlactating (NP/NL) states. Superscripts indicate data from three individuals who were sampled repeatedly. * Not applicable.

Season	Age category	Sex	Reproductive state	Body mass (g)	HCC (pg/mg)	Individual ID
Wet	Adult	F	NP/NL	250	794.12	M16
			NP/NL	275	789.23	M17
			P/L	308	535.58	M26
			P/L	320	2,007.78	M32
			NP/NL	290	417.08	M38
			NP/NL	276	677.01	M39
			P/L	297	657.32	M42
			P/L	294	689.13	M44
			NP/NL	279	1,436.53	M51^1^
		M	*	280	328.20	M12
			*	250	366.22	M15
			*	284	1,319.93	M31
			*	245	704.21	M40
			*	244	619.58	M41
			*	282	531.38	M43
			*	288	246.51	M48
			*	264	702.84	M49^1^
	Subadult	M	*	241	1,671.80	M33
	Juvenile	F	*	160	3,491.93	M34
			*	169	2,259.61	M35^1^
			*	162	966.97	M45
		M	*	190	1,181.82	M13
			*	200	1,518.40	M14
			*	120	1,933.15	M18
			*	110	2,084.69	M19
			*	172	1,029.87	M46
			*	119	3,246.44	M47
Dry	Adult	F	P/L	281	817.90	M51^2^
			P/L	357	966.97	M53
			P/L	313	2,741.55	M51^3^
			P/L	315	4,295.46	M63
			P/L	294	2,619.59	M65
			NP/NL	262	997.26	M70
			P/L	297	2,889.62	M72
			NP/NL	294	1,876.08	M73
			NP/NL	247	2,061.67	M81
		M	*	260	933.43	M22
			*	270	231.02	M23
			*	280	970.74	M25
			*	255	631.93	M49^2^
			*	275	686.39	M52
			*	286	835.84	M54
			*	284	466.64	M57
			*	284	1,177.37	M49^3^
			*	284	2,073.61	M64
			*	257	1,021.90	M66
			*	257	585.23	M71
			*	313	1,392.48	M74
			*	271	2,011.95	M76
			*	308	1,671.94	M79
			*	265	2,045.73	M80
	Subadult	M	*	215	852.42	M27
			*	233	1,092.49	M28
			*	247	1,959.06	M75
	Juvenile	F	*	234	2,113.26	M35^2^
			*	168	3,024.24	M56
			*	162	3,523.09	M58
			*	159	2,793.13	M67
			*	189	1,583.09	M69
			*	183	2,900.54	M82
			*	198	2,808.69	M83
		M	*	172	1,419.66	M24
			*	140	2,725.76	M59
			*	141	2,541.69	M62
			*	139	2,674.82	M77
			*	125	4,088.90	M78

Contrary to our prediction, sex was found to influence HCC, *F* (1, 57) = 4.95, *p* = 0.03, η_p_^2^ = 0.080, with females showing higher HCC (*M* = 1823, SE = 197) than males (*M* = 1379, SE = 151; Fig. 1C). All interactions were non-significant and irrelevant to our hypotheses, all *F* < 2.357, *p* ≥ 0.13, η_p_^2^ ≤ 0.040.

We next evaluated whether there was an effect of reproductive condition and season on HCC in adult females. Body mass was not a significant covariate in this analysis, *F* (1, 12) = 0.36, *p* = 0.056, η_p_^2^ = 0.086. Female reproductive condition did not significantly affect HCC, *F* (1, 12) = 1.260, *p* = 0.284, η_p_^2^ = 0.179. However, HCC did vary by season in adult females, with HCC higher in the dry season (*M* = 2107.09, SE = 286.6) than the wet season (*M* = 933.53, SE = 126.9), *F* (1, 12) = 4.292, *p* = 0.061, η_p_^2^ = 0.478 (Fig. 1D).

Finally, during our study we documented 21 birth events involving eight common marmoset females. The number of litters per female ranged from one to five and the number of infants we observed was 39. We do not know whether all births produced twins (total 42 infants) and in three cases one of the twins died shortly after birth (a few days or 1–2 weeks) or whether three of the 21 births produced singletons. Twenty-nine of the infants survived past weaning (4 months of age), resulting in 25–30% infant mortality. Based on group censuses and retrapping, we found that 15 of the surviving 29 offspring were present in their natal group by 1 year of age (end of juvenile period). Therefore, assuming that juveniles who disappeared from their natal group had died, we estimated that juvenile mortality in this CAT marmoset population was 48%.

## Discussion

In this study, we examined HCC in wild adult female, adult male, subadult and juvenile common marmosets naturally inhabiting an extremely hot and dry region of northeastern Brazil. Across the 3 years of our study, rainfall varied from only 177–412 mm per year, with 64–72% of all precipitation occuring during a 3 month period. During the months of October through April, temperatures at our field site reached or exceeded 33 °C on 65–86% of days (Instituto Nacional de Meteorologia-INMET).

Our most important findings were that across all age and sex classes, HCC was higher in the dry season compared with the wet season, female reproductive condition (pregnant and lactating *vs.* not reproductively active) did not predict HCC, adult females had higher HCC than adult males, and juveniles had higher HCC than adults. Elevated HCC in CAT common marmosets during the dry season appears to represent a physiological response to an extended period of heat, water and, possibly, nutritional stress. The majority of CAT trees are deciduous during the dry season. During this period, fleshy fruits and insects, which account for over 50% of yearly marmoset feeding time, are in limited supply (Amora, Beltrão-Mendes & Ferrari, 2013). CAT marmosets respond to dry season conditions by devoting almost 20% of their total daily activity budget to exploiting exudates (gums and saps, a renewable resource), increasing their dependance on cactus flesh as a reliable source of water and nutrients, resting during the hottest times of the day, seeking refuge in shaded rock crevices, and licking the dew that collected on leaves in the early morning (Abreu et al., 2016; De la Fuente et al., 2014; Garber et al., 2019). In addition, compared to Atlantic Forest common marmosets, adult Caatinga common marmosets weight 11–20% less, and exhibit a higher surface area relative to body mass, which may play an important role in dissipating body heat in order to maintain thermal homeostasis (Garber et al., 2019). The fact that both males and females exhibited significantly higher HCC in the dry season compared to the wet season, lends support to our hypothesis that during this 5–8 month period of the year, CAT marmosets are characterized by increased levels of ongoing physiological stress.

### Effects of sex and reproduction on HCC

A second important finding was that, unrelated to season, female reproductive condition (pregnancy/lactation *vs.* nonpregnant/nonlactating) did not significantly affect HCC in common marmosets. Given their potentially high reproductive output and the energetic costs of reproduction (production of twin offspring, ability to produce two litters per year, combined weight of twins at birth representing 15–20% of maternal weight and increased water intake required to nurse twin infants (Garber & Leigh, 1997), we expected breeding females to have elevated HCC compared with nonbreeding females. However, this was not the case. The fact that adult females, regardless of reproductive condition, were found to have significantly higher HCC compared with adult males suggests that wild female common marmosets face a combination of social and environmental stressors associated with competitive interactions resulting in infertility or subfertility in subordinate adult females (Saltzman et al., 2008) and high reproductive output and reproductive sovereignty in breeding females, that differ from stress loads encountered by males, and contribute to elevated HCC. We note that Phillips et al. (2018) found no evidence of adult sex-based differences in HCC in captive common marmosets. Her groups, however, were housed in non-currently reproducing family groups containing a single adult male and single adult female. The absence of stressors associated with female reproductive competition and the nutritional costs of producing twin infants in this captive population, may help to explain sex differences in HCC present in our wild marmoset population but absent in captive individuals.

### Elevated HCC in juveniles

Our finding that cortisol decreased with age in common marmosets is consistent with reports from several wild and captive primate populations (Fourie & Bernstein, 2011; Gesquiere et al., 2005; Pryce, Palme & Feldon, 2002). If elevated cortisol in juveniles compared with adults is solely a function of age-specific differences in physiology (i.e., elevated in juveniles in response to lower corticosteroid binding globulin concentration), then it is not a true indicator of increased physiological stress, nutrient and/or water deficiency, or decreased health. However, HCC was found to correlate negatively with survivorship in a population of wild gray mouse lemurs (*Microcebus murinus*; (Rakotoniaina et al., 2017). In this species, survival rates of juveniles during the winter were 19–40% lower compared with those of adults (Kraus, Eberle & Kappeler, 2008). Rakotoniaina et al. (2017) argued that HCC in gray mouse lemurs “may underlie demographic fluctuations of natural populations.”

In the case of common marmosets, AF and CAT populations differ in certain critical aspects of demography that may provide an ecological explanation for the consequences of elevated HCC in juveniles. In the CAT, despite the fact that females commonly produce two to four infants per year, group size averages only 6 ± 1 individuals, including three to four adults (based on 13 groups see Table SII in Garber et al., 2019). Our largest CAT group totaled nine individuals. In contrast, although female AF common marmosets also produce two to four infants per year, group size averages 9 ± 3 individuals (including an average of five adults), with the largest groups containing 16 individuals (Table SII in Garber et al., 2019). Therefore, we asked the question, how can CAT marmosets maintain such small group sizes if the breeding female produces two to four offspring per year.

Two factors that affect group size are infant mortality and juvenile mortality. Infant mortality in both AF and CAT common marmoset populations averages 25–30% (Garber et al., 2019). Although we lack quantitative data on juvenile mortality in AF common marmosets, 48% (14/29) of the juveniles in our CAT population disappeared from their natal groups. Given that each was less than 1 year of age when they disappeared, and that during the 3 years of our study no juvenile was observed to immigrate into any of our six focal groups, we argue that the most plausible explanation is that these immature individuals did not survive the juvenile period. This is supported by demographic data on a close relative of common marmosets, the Amazonian saddle back tamarins (*Leontocebus weddelli*, formerly *Saguinus fuscicollis weddelli*). In Weddell’s saddleback tamarin, individuals migrate from their natal groups between 20 and 45 months of age (Goldizen et al., 1996). Mortality to 1.5 years of age in this tamarin population was 14% (9 of 63 natal individuals), which is considerably lower than in our CAT marmoset population.

Mortality risk during juvenility, a period during which young individuals have attained locomotor and dietary independence but are not yet fully adult-like in body mass, social skills and cognitive development, has been a focus of primate life history theory (Leigh & Blomquist, 2011). And although quantitative data on juvenile risk in wild primates are extremely limited, it is assumed that factors such as predation, disease, and/or reduced competitiveness in obtaining access to high quality resources are the primary drivers of juvenile mortality (Janson & van Schaik, 1993). Our CAT field site contains at least seven potential marmoset predators including two species of raptors (burrowing owls, *Athene cunicularia;* Southern crested caracaras, *Caracara plancus*), two species of predatory snakes (boas, *Boa constrictor*; rattlesnakes, *Crotalus* sp.) and three species of carnivores (oncillas, *Leopardus triginus;* Crab-eating foxes, *Cerdocyon thous*; Domesticated dogs, *Canis familiaris*). Each of these predators are present year-round (Cardoso da Silva, Leal & Tabarelli, 2017; Passos Filho et al., 2015). Moreover, we note that compared with other taxa of small New World monkeys (e.g., squirrel monkeys, titi monkeys and night monkeys), immature tamarins and marmosets are characterized by an extended period of dietary dependance and a pattern of delayed brain growth (Garber & Leigh, 1997) during which adult helpers provision the young with fruits and insects well into the juvenile period (Ferrari, 1992; Schiel et al., 2010). What is less clear, however, is the degree to which the transition from provisioning to dietary independence represents a critical period in juvenile survival, especially in a hot, dry and food-limited environment like the CAT. Elevated HCC in juvenile CAT marmosets, therefore, may represent an honest indicator of environmental stress that is consistent with the high level of juvenile mortality in our study population. However, additional data, including quantitative information on changes in food availability and distribution during the wet and dry seasons, mortality rate, and HCC, are necessary to evaluate this hypothesis.

## Conclusion

Common marmosets living in the harsh Brazilian Caatinga, which is characterized by an extended hot and dry season and low primary productivity, experienced increased levels of chronic and/or nutritional stress as reflected in HCC. Juveniles had higher HCC than adults; and regardless of age or sex, and individuals experienced higher HCC during the dry season compared with the wet season. Given the set of environmental stressors present in the Caatinga (heat, water, and food stress, especially during the dry season and year-round predation risk), we suggest the high levels of HCC found in juveniles is an indicator of chronic stress and coincides with high rates of juvenile mortality that characterize this population.

## Additional Information and Declarations

### Data Availability

The following information was supplied regarding data availability: The hair cortisol concentration for each individual and independent variables sex, age group, and season are available in Table 1.

## References

[ref-1] Abreu F, De la Fuente MF, Schiel N, Souto A (2016). Feeding ecology and behavioral adjustments: flexibility of a small neotropical primate (*Callithrix jacchus*) to survive in a semiarid environment. Mammal Research.

[ref-2] Amora TT, Beltrão-Mendes R, Ferrari SF (2013). Use of alternative plant resources by common marmosets (*Callithrix jacchus*) in the semi-arid Caatinga scrub forests of Northeastern Brazil. American Journal of Primatology.

[ref-3] Araújo EL, Castro CC, Alburquerque UP (2007). Dynamics of Brazilian Caatinga—a review concerning the plants, environment and people. Functional Ecosystems and Communities.

[ref-4] Bales KL, French JA, Hostetler CM, Dietz JM (2005). Social and reproductive factors affecting cortisol levels in wild female golden lion tamarins (*Leontopithecus rosalia*). American Journal of Primatology.

[ref-5] Bales KL, French JA, McWilliams J, Lake RA, Dietz JM (2006). Effects of social status, age, and season on androgen and cortisol levels in wild male golden lion tamarins (*Leontopithecus rosalia*). Hormones and Behavior.

[ref-6] Bechshoft TO, Sonne C, Riget FF, Letcher RJ, Novak MA, Henchey E, Meyer JS, Eulaers I, Jaspers VLB, Covaci A, Dietz R (2013). Polar bear stress hormone cortisol fluctuates with the North Atlantic Oscillation climate index. Polar Biology.

[ref-7] Bruener CW, Delehanty B, Boonstra R (2013). Evaluating stress in natural populations of vertebrates: total CORT is not good enough. Functional Ecology.

[ref-8] Bryan HM, Darimont CT, Paquet PC, Wynne-Edwards KE, Smits JEG (2013). Stress and reproductive hormones in brown bears reflect nutritional benefits and social consequences of a salmon foraging niche. PLOS ONE.

[ref-9] Buckner JC, Lynch Alfaro JW, Rylands AB, Alfaro ME (2015). Biogeography of the marmosets and tamarins (Callitrichidae). Molecular Phylogenetics and Evolution.

[ref-10] Cardoso da Silva JM, Leal IR, Tabarelli M (2017). Caatinga: the largest tropical dry forest region in South America.

[ref-11] Carlitz EHD, Kirschbaum C, Stalder T, Van Schaik CP (2014). Hair as a long-term retrospective cortisol calendar in orang-utans (*Pongo* spp.): new perspectives for stress monitoring in captive management and conservation. General and Comparative Endocrinology.

[ref-12] Carlitz EHD, Miller R, Kirschbaum C, Gao W, Hänni C, van Schaik CP (2016). Measuring hair cortisol concentrations to assess the effect of anthropogenic impacts on wild chimpanzees (*Pan troglodytes*). PLOS ONE.

[ref-13] Cattet M, MacBeth BJ, Janz DM, Zedrosser A, Swenson JE, Dumond M, Stenhouse GB (2014). Quantifying long-term stress in brown bears with the hair cortisol concentration: a biomarker that may be confounded by rapid changes in response to capture and handling. Conservation Physiology.

[ref-14] Cavigelli SA (1999). Behavioural patterns associated with faecal cortisol levels in free-ranging female ring-tailed lemurs, Lemur catta. Animal Behaviour.

[ref-15] De la Fuente MF, Schiel N, Bicca-Marques JC, Caselli CB, Souto A, Garber PA (2019). Balancing contest competition, scramble competition, and social tolerance at feeding sites in wild common marmosets (*Callithrix jacchus*). American Journal of Primatology.

[ref-16] De la Fuente MF, Souto A, Sampaio MB, Schiel N (2014). Behavioral adjustments by a small neotropical primate (*Callithrix jacchus*) in a semiarid Caatinga environment. The Scientific World Journal.

[ref-17] Dettmer AM, Novak MA, Meyer JS, Suomi SJ (2014). Population density-dependent hair cortisol concentrations in rhesus monkeys (*Macaca mulatta*). Psychoneuroendocrinology.

[ref-18] Fardi S, Sauther ML, Cuozzo FP, Jacky IAY, Bernstein RM (2018). The effect of extreme weather events on hair cortisol and body weight in a wild ring-tailed lemur population (*Lemur catta*) in southwesten Madagascar. American Journal of Primatology.

[ref-19] Ferrari SF (1992). The care of infants in a wild marmoset (*Callithrix flaviceps*) group. American Journal of Primatology.

[ref-20] Fourie NH, Bernstein RM (2011). Hair cortisol levels track phylogenetic and age related differences in hypothalamic–pituitary–adrenal (HPA) axis activity in non-human primates. General and Comparative Endocrinology.

[ref-21] Fourie NH, Jolly CJ, Phillips-Conroy JE, Brown JL, Bernstein RM (2015a). Variation of hair cortisol concentrations among wild populations of two baboons species (*Papio anubis*, *P. hanadryas*) and a population of their natural hybrids. Primates.

[ref-22] Fourie NH, Turner TR, Brown JL, Pampush JD, Lorenz JG, Bernstein RM (2015b). Variation in vervet (*Chlorocebus aethiops*) hair cortisol concentrations reflects ecological disturbance by humans. Primates.

[ref-23] Garber PA (1997). One for all and breeding for one: cooperation and competition as a tamarin reproductive strategy. Evolutionary Anthropology.

[ref-24] Garber PA, Caselli CB, McKenney AC, Abreu F, De la Fuente MF, Araújo A, Arruda MF, Souto A, Schiel N, Bicca-Marques JC (2019). Trait variation and trait stability in common marmosets (*Callithrix jacchus*) inhabiting ecologically distinct habitats in northeastern Brazil. American Journal of Primatology.

[ref-25] Garber PA, Leigh S (1997). Ontogenetic variation in small-bodied New World primates: implications for patterns of reproduction and infant care. Folia Primatologica.

[ref-26] Garber PA, Porter LM, Spross J, Di Fiore A (2016). Tamarins: insights into monogamous and non-monogamous single female social and breeding systems. American Journal of Primatology.

[ref-27] Gesquiere LR, Altmann J, Khan MZ, Couret J, Yu JC, Endres CS, Lynch JW, Ogola P, Fox EA, Wango EO, Alberts SC (2005). Coming of age: steroid hormones of wild immature baboons (*Papio cynocephalus*). American Journal of Primatology.

[ref-28] Ghassemi Nejad J, Lohakare JD, Son JK, Kwon EG, West JW, Sung KI (2014). Wool cortisol is a better indicator of stress than blood cortisol in ewes exposed to heat stress and water restriction. Animal.

[ref-29] Goldizen AW, Mendelson J, van Vlaardingen M, Terborgh J (1996). Saddle-back tamarin (*Saguinus fuscicollis*) reproductive strategies: evidence from a thirteen-year study of a marked population. American Journal of Primatology.

[ref-30] Gonzalez-De-la-Vara Md R, Valdez RA, Lemus-Ramirez V, Vazquez-Chagoyan JC, Villa-Godoy A, Romano MC (2011). Effects of adrenocorticotropic hormone channenge and age on hair cortisol concentrations in dairy cattle. Canadian Journal of Veterinary Research = Revue canadienne de recherche veterinaire.

[ref-31] Grant KS, Worlein JM, Meyer JS, Novak MA, Kroeker R, Rosenberg K, Kenney C, Burbacher TM (2017). A longitudinal study of hair cortisol concentrations in *Macaca nemestrina* mothers and infants. American Journal of Primatology.

[ref-32] Greff MJE, Levine JM, Abuzgaia AM, Elzagallaai AA, Rieder MJ, Van Uum SHM (2019). Hair cortisol analysis: an update on methodlogical considerations and clinical applications. Clinical Biochemistry.

[ref-33] Heimburge S, Kanitz E, Otten W (2019). The use of hair cortisol for the assessment of stress in animals. General and Comparative Endocrinology.

[ref-34] Hershkovitz P (1977). Living New World Monkeys (Platyrrhini).

[ref-35] Janson CH, van Schaik CP, Pereira ME, Faurbanks LA (1993). Ecological risk aversion in juvenile primates: slow and steady wins the race. Juvenile Primates: Life History, Development and Behavior.

[ref-36] Kalliokoski O, Jellestad FK, Murison R (2019). A systematic review of studies utilizing hair glucocorticoids as a measure of stress suggests the marker is more appropriate for quantifying short-term stressors. Scientific Reports.

[ref-37] Katsu Y, Iguchi T (2016). Subchapter 95D—Cortisol.

[ref-38] Khansari DN, Murgo AJ, Faith RE (1990). Effects of stress on the immune-system. Immunology Today.

[ref-39] Koenig A (1995). Group size, composition, and reproductive success in wild common marmosets (*Callithrix jacchus*). American Journal of Primatology.

[ref-40] Kraus C, Eberle M, Kappeler PM (2008). The costs of risky male behaviour: sex differences in seasonal survival in a small sexually monomorphic primate. Proceedings of the Royal Society B: Biological Sciences.

[ref-41] Laudenschlager ML, Jorgensen MJ, Fairbanks LA (2012). Developmental patterns of hair cortisol in male and female nonhuman primates: lower hair cortisol levels in vervet males emerge at puberty. Psychoneuroendocrinology.

[ref-42] Leigh SR, Blomquist GE, Campbell CJ, Fuentes A, MacKinnon KC, Bearder SK, Stumpf RM (2011). Life history. Primates in Perspective.

[ref-43] McEwen BS (1998). Protective and damaging effects of stress mediators. New England Journal of Medicine.

[ref-44] Meyer J, Novak M (2012). Minireview: hair cortisol: a novel biomarker of hypothalamic–pituitary–adrenocortical activity. Endocrinology.

[ref-45] Meyer J, Novak M, Hamel A, Rosenberg K (2014). Extraction and analysis of cortisol from human and monkey hair. Journal of Visualized Experiments.

[ref-46] Padgett DA, Glaser R (2003). How stress influences the immune response. Trends in Immunology.

[ref-47] Parker AJ, Hamlin GP, Coleman CJ, Fitzpatrick LA (2003). Dehydration in stressed ruminants may be the result of acortisol-induced diuresis. Journal of Animal Science.

[ref-48] Passos Filho PB, Chaves LS, Carvalho RA, Alves PP, Dassunção MM (2015). Fauna ilustrada da fazenda tamanduá.

[ref-49] Phillips KA, Tukan AN, Rigodanzo AD, Reusch RT, Brasky KM, Meyer JS (2018). Quantification of hair cortisol concentration in common marmosets (*Callithrix jacchus*) and tufted capuchins (*Cebus apella*). American Journal of Primatology.

[ref-50] Pryce CR, Palme R, Feldon J (2002). Development of pituitary-adrenal endocrine function in the marmoset monkey: infant hypercortisolism is the norm. Journal of Clinical Endocrinology & Metabolism.

[ref-51] Rakotoniaina JH, Kappeler PM, Kaesler E, Hämäläinen AM, Kirschbaum C, Kraus C (2017). Hair cortisol concentrations correlate negatively with survival in a wild primate population. BMC Ecology.

[ref-52] Russell E, Koren G, Rieder MJ, van Uum SHM (2012). Hair cortisol as a biological marker of chronic stress: current status, future directions and unanswered questions. Psychoneuroendocrinology.

[ref-53] Rylands AB, Coimbra-Filho AF, Mittermeier RA, Ford SM, Porter LM, Davis LC (2009). The systematics and distributions of marmosets (*Callithrix, Callibella, Cebuella*, and *Mico*). The Smallest Anthropoids: The Marmoset/Callimico Radiation.

[ref-54] Saltzman W, Liedl KJ, Salper OJ, Pick RR, Abbott DH (2008). Post-conception eproductive competition in cooperatively breeding common marmosets (*Callithrix jacchus*). Hormones and Behavior.

[ref-55] Sanin YL, Zuluaga Cabrera AM, Tarazona Morales AM (2015). Adaptive responses to thermal stress in mammals. Revue de Médecine Vétérinaire.

[ref-56] Schiel N, Souto A (2017). The common marmoset: an overview of its natural history, ecology and behavior. Developmental Neurobiology.

[ref-57] Schiel N, Souto A, Huber L, Bezerra BM (2010). Hunting strategies in wild common marmosets are prey and age dependent. American Journal of Primatology.

[ref-58] Seltmann A, Czirjak GA, Courtiol A, Bernard H, Struebig MJ, Voigt CC (2017). Habitat disturbance results in chronic stress and impaired health status in forest-dwelling paleotropical bats. Conservation Physiology.

[ref-59] Strasser EH, Heath JA (2013). Reproductive failure of a human-tolerant species, the American Kestrel, is associated with stress and human disturbance. Journal of Applied Ecology.

[ref-60] Takahashi M (2012). Heat stress on reproductive function and fertility in mammals. Reproductive Medicine and Biology.

[ref-61] Tecot SR, Masters J, Gamba M, Genin F (2013). Variable energetic strategies in disturbed and undisturbed rain forest: *Eulelemur rubiventer* fecal cortisol levels in South-Eastern Madagascar. Leaping Ahead: Advances in Prosimian Biology.

[ref-62] Weingrill T, Gray DA, Barrett L, Henzi SP (2004). Fecal cortisol levels in free-ranging female chacma baboons: relationship to dominance, reproductive state and environmental factors. Hormones and Behavior.

[ref-63] Yamamoto ME, Arruda MF, Alencar AI, Sousa MBC, Araújo A, Ford SM, Porter LM, Davis LC (2009). Mating systems and female-female competition in the common marmoset (*Callithrix jacchus*). The Smallest Anthropoids: The Marmoset/Callimico Radiation.

[ref-64] Yamanashi Y, Morimura N, Mori Y, Hayashi M, Suzuki J (2013). Cortisol analysis of hair of captive chimpanzees (*Pan troglodytes*). General and Comparative Endocrinology.

[ref-65] Yamanashi Y, Teramoto M, Morimura N, Hirata S, Suzuki J, Hayashi M, Kinoshita K, Murayama M, Idani G’ichi (2016). Analysis of hair cortisol levels in captive chimpanzees: Effect of various methods on cortisol stability and variability. MethodsX.

